# Predicting health outcomes with intensive longitudinal data collected by mobile health devices: a functional principal component regression approach

**DOI:** 10.1186/s12874-024-02193-7

**Published:** 2024-03-17

**Authors:** Qing Yang, Meilin Jiang, Cai Li, Sheng Luo, Matthew J. Crowley, Ryan J. Shaw

**Affiliations:** 1 School of Nursing, Duke University, Durham, USA; 2 Eli Lilly and Company, Indianapolis, IN USA; 3 Department of Biostatistics, St. Jude Children’s Research Hospital, Memphis, TN USA; 4 Biostatistics & Bioinformatics, Duke University, Durham, USA; 5 Center of Innovation to Accelerate Discovery and Practice Transformation, Durham Veterans Affairs Medical Center, Durham, NC USA; 6 Division of Endocrinology, Diabetes and Metabolism, Duke University School of Medicine, Durham, NC USA; 7 Center for Applied Genomics & Precision Medicine, School of Medicine, Duke University, Durham, NC USA

## Abstract

**Background:**

Intensive longitudinal data (ILD) collected in near real time by mobile health devices provide a new opportunity for monitoring chronic diseases, early disease risk prediction, and disease prevention in health research. Functional data analysis, specifically functional principal component analysis, has great potential to abstract trends in ILD but has not been used extensively in mobile health research.

**Objective:**

To introduce functional principal component analysis (fPCA) and demonstrate its potential applicability in estimating trends in ILD collected by mobile heath devices, assessing longitudinal association between ILD and health outcomes, and predicting health outcomes.

**Methods:**

fPCA and scalar-to-function regression models were reviewed. A case study was used to illustrate the process of abstracting trends in intensively self-measured blood glucose using functional principal component analysis and then predicting future HbA1c values in patients with type 2 diabetes using a scalar-to-function regression model.

**Results:**

Based on the scalar-to-function regression model results, there was a slightly increasing trend between daily blood glucose measures and HbA1c. 61% of variation in HbA1c could be predicted by the three preceding months’ blood glucose values measured before breakfast (*P* < 0.0001, ${R}_{adjusted}^{2}=0.61$).

**Conclusions:**

Functional data analysis, specifically fPCA, offers a unique tool to capture patterns in ILD collected by mobile health devices. It is particularly useful in assessing longitudinal dynamic association between repeated measures and outcomes, and can be easily integrated in prediction models to improve prediction precision.

**Supplementary Information:**

The online version contains supplementary material available at 10.1186/s12874-024-02193-7.

## Introduction

Owing to the ubiquity of smartphones and Bluetooth devices in the consumer market, coupled with fast-developing mobile health technologies, health data have become easily captured, stored, and accessed [1]. This new mode of device data, usually referred to as intensive longitudinal data (ILD) [2], can be measured tens, hundreds, and even thousands of times within a specific time interval, such as hour, day, or month. Compared to traditional clinical measurements at small numbers of discrete clinic visits and panel surveys, mobile health device generated ILD can capture trends in data at a more granular level. This abundance of data availability in near-real time provides tremendous opportunity for disease monitoring, early risk prediction and prevention in healthcare [3]. Specifically, as self-monitoring between clinic visits is essential for managing chronic disease such as type 2 diabetes and hypertension [1, 4], many patients use mobile health devices to collect and self-monitor various health indicators and health behaviors on a daily basis over a long time period. There is an emerging need to use these intensively collected data to support patients with chronic illnesses in managing their conditions between clinic visits.

While a variety of mobile health technologies may facilitate data collection, there are considerable challenges in managing and analyzing the ILD they generate. Specifically, due to singularity issue when the number of repeated measurements is more than the number of participants, standard regression models may not allow coefficients to be estimated uniquely [4]. A simple and traditional way to handle ILD is the response feature approach, in which data are summarized either by a single summary statistic (i.e., mean or median) or several repeated summary statistics over certain time windows, such as averaging measurements by week or month [2]. Then the data can be analyzed using linear model or linear mixed models. However, this approach would result in the loss of information, and there is no clear evidence to support what time interval is meaningful to use for summary statistics. Therefore, a better way to analyze intensive longitudinal data while retaining most of its value is needed.

A prominent feature of ILD is that they are often in a continuous-time nature that can be inherently represented by an underlying curve, a stochastic process, or a function over time. For instance, although a patient with diabetes typically measures their blood glucose level several times a week, the values can exist at any time within the period and can be considered as functional data. Functional data analysis (FDA) is a class of statistical approaches specially designed to represent the data structure (underlying smooth curve) in ILD that summarizes the trend using a small number of variables [5]. Specifically, functional principal component analysis (fPCA), an emerging first-line approach in FDA, has been used to recover individual complex trajectories [6–12], and to cluster patients based on their distinct trajectory patterns [13–15]. Functional regression modelling, which uses functional data as covariates through fPCA, was developed to explore the longitudinal association between ILD and a scalar outcome [16]. While offering a promising statistical tool to extract trend information in ILD for assessing longitudinal association and conducting risk prediction [17], the application of this method in mobile health research is scarce due to the complexity and relative unfamiliarity of FDA.

This paper serves as a timely and practical guide to illustrate the use of the functional regression model in assessing longitudinal relationship between ILD and health outcome, making risk predictions and recovering individual trajectories. We provide a brief introduction to the functional regression model and available statistical software for conducting this analysis. We then provide an illustrative example to demonstrate the functional regression analysis process step by step using data collected from a mobile health study with type II diabetes patients.

### Functional Data Analysis (FDA) and functional regression model

The concept of functional data and the use of functional data analysis for ILD were introduced by Ramsay & Silverman [18, 19]. Although ILD is discretely measured, they can be considered as functional data because the true values are continuous over a time interval and are regulated by an underlying smooth curve or a function. The basic idea for FDA is to extract trend information from the ILD and construct functional curves for each subject using a linear combination of small numbers of functions through a variety of statistical methods and techniques, including basis expansion and roughness penalty. Various dimension reduction methods can then be applied to the functional object with fPCA being one of the most used due to its flexibility. fPCA is an extension of standard principal component analysis [20] in the functional space. While PCA handles multivariate data as discrete observations, which suits cross-sectional data, fPCA models data as a stochastic process which is smooth trajectories other than discrete data points, which is better for longitudinal data [21]. Indeed, this approach is particularly well-suited to our ILD data, as it enables us to model the latent trajectory of blood glucose levels across a specific time frame. Such modeling offers valuable insights into the dynamic relationship between these levels and health outcomes as time progresses. Conceptually speaking, fPCA captures the variations in functional/longitudinal data by using a few functions over time weighted by uncorrelated variables. After the dimension reduction of ILD to a linear combination of a few functional principal components, they could be used as outcome (functional response model) or predictor (scalar-on-function regression model) or both (function-on-function regression model). An excellent review of all types of functional regression model using fPCA is provided in the books for functional data analysis [16, 22–24]. In this section, we will focus on using scalar-on-function functional regression model [25] to study the association between ILD and a scalar outcome. The model is formulated as

1 $${Y_i} = \alpha + \int {{X_i}} \left( t \right)\beta \left( t \right)dt + {\epsilon _i},$$

where $\alpha$ is the intercept, $\beta \left(t\right)$ is the coefficient function of time t, which indicates level of importance of each measurement over time with respect to scalar outcome $Y$, and ${{\epsilon}_{i}}$ is the random error that follows the distribution of $N(0, {\sigma }^{2})$, $i=1,\dots , n$. The biggest difference compared to regular linear regression is that both the regressor ${X}_{i}\left(t\right)$ and coefficient function $\beta \left(t\right)$ are functions of time t. There are different ways to obtain unique estimation for $\beta \left(t\right)$ and fPCA-based method is the most commonly used one. The estimation process is conducted in two stages.

In the first stage, we need to represent intensively measured longitudinal data by smooth random functions ${X}_{i}\left(t\right)$. The fPCA approach models the data as smooth covariance functions with respect to different time points. The dimension of ILD is usually large given the large number of time points, and the correlations between these repeated measurements are high. fPCA uses *Karhunen*–*Loève* expansion to abstract orthogonal functions which represent the most prominent trends in variation of data. For the i^th^ person, assume that the ILD have been centered [16, 26–29], then the underlying trajectory ${X}_{i}\left(t\right)$ can be approximated by

2 $${X}_{i}\left(t\right)\approx \sum _{j=1}^{p}{\widehat{\zeta }}_{ij}{\widehat{\upsilon }}_{j}\left(t\right),$$

where ${\widehat{\upsilon }}_{j}\left(t\right)$ is the j^th^ estimated eigenfunction or estimated functional principal component (EFPC) of the covariance function of $X\left(t\right)$ among top $p$ important EFPCs, and ${\widehat{\zeta }}_{ij}$ is the corresponding j^th^ estimated random score of i^th^ person, which is assumed to follow an independent and identically distributed (i.i.d.) normal distribution. The first component ${\upsilon }_{1}\left(t\right)$ represents the most significant trend deviated from the mean function since it explains the largest portion of variance. The score ${\zeta }_{ij}$ associated with each component describes how much ${\upsilon }_{j}\left(t\right)$ contributes to the i^th^ person’s subject-specific deviation from population mean function. Throughout the paper, the hat over a parameter indicates the parameter or function estimate.

After representing ${X}_{i}\left(t\right)$ as a few principal components, in the second stage, we can proceed to the regression model part. It is assumed that the coefficient function $\beta \left(t\right)$ in Eq. (1) can be expanded by eigenfunctions such that

3 $$\beta \left(t\right)=\sum _{j=1}^{p}{\beta }_{j}{\upsilon }_{j}\left(t\right)$$.

Replacing ${X}_{i}\left(t\right)$ by a set of smooth curves according to (2), the regression model in Eq. (1) becomes a regular linear regression model shown as below

$${Y_i} = \alpha + \int \beta \left( t \right)\left( {\sum\limits_{j = 1}^p {{{\hat \zeta }_{ij}}} {{\hat \upsilon }_j}\left( t \right)} \right)dt + {\epsilon _i},$$

4 $$= \alpha + \sum\limits_{j = 1}^p {{{\hat \zeta }_{ij}}} {\beta _j} + {\epsilon _i},$$

where ${\widehat{\zeta }}_{ij}$ is the functional score that was estimated from (2) and can be treated as the pseudo-covariates after dimension reduction. $\alpha$ is the intercept and ${\beta }_{j}=\int \beta \left(t\right){\widehat{\upsilon }}_{j}\left(t\right)dt$ is the estimated coefficient for the j^th^ component. Similar to a regular linear regression, we can obtain estimated intercept $\widehat{\alpha }$ and coefficient for each component ${\widehat{\beta }}_{j}$by least square estimates. We then use the estimated coefficients ${\widehat{\beta }}_{j}$ in Eq. (3) to compute the original coefficient function $\widehat{\beta }\left(t\right)$ as follows:

5 $$\widehat{\beta }\left(t\right)=\sum _{j=1}^{p}{\widehat{\beta }}_{j}{\widehat{\upsilon }}_{j}\left(t\right)$$.

More detailed modeling and estimation steps can be found in the supplemental materials.

Commonly used estimation methods for fPCA include smoothing or imputation approaches [5]. Missing data can be handled by either removing records that containing missing values or apply missing data imputation. When there is a large amount of missing data, or when the repeated measures are noisy or at irregular time points, fPCA for sparse functional data can be used. This method can borrow information across samples and produce a more stable and accurate estimation [30, 31].

Several statistical software is readily available for FDA. The R and MATLAB package “fda” [26] were as first developed to implement basic tools of FDA, and the “refund” R package [32] was built to provide more flexible and advanced functional models like various functional regression models. In addition, the “face” package [33] was specially designed to conduct fPCA for sparse functional data or longitudinal data. Recently, the R package “mfaces” [34] was developed to advance multivariate fPCA for multiple sparse functional data. In our illustrative example, we will implement the fPCA using the “face” package in R.

### An empirical example: functional PCA regression model using intensive mobile health data

As an illustrative example, we built a scalar-on-function regression model using data from an observational study that was designed to explore the feasibility of using multiple mobile health devices to facilitate patients’ self-management for their type 2 diabetes mellitus [35]. While blood glucose is an important measure for day-to-day management, HbA1c reflects the average blood glucose levels over the past 2–3 months, offering a more stable and comprehensive view of blood sugar control. Furthermore, HbA1c is the only measure of glycemia that has been studied as a means to predict long-term microvascular and macrovascular diabetes complications. Thus, HbA1c remains the single most important glycemic measure for providers and patients alike. Although Hemoglobin A1c (HbA1c) is the main health indicator for type 2 diabetes mellitus patients, patients usually need to visit clinics and have HbA1c checked in a lab every 3–6 months [36]. Between clinic visits, patients were asked to monitor their blood glucose using a glucometer at least on a weekly basis. While there is a suggested controlled range for blood glucose, blood glucose does fluctuate widely based on time of measurement, diet, and other factors [37]. Although a calculator is available to convert average blood sugar to HbA1c, patients may find it challenging to calculate their average blood sugar accurately. According to a recently conducted qualitative research study, patients expressed prefererence for receiving projections of their HbA1c every time they input the self-measured blood glucose measures from a glucometer [38]. Ideally, it would be more convenient to develop a prediction model that could be incorporated in the mobile device to predict HbA1c based on all the input glucometer readings for patients. Additionally, we know that HbA1c reflects red blood cell turnover, which typically occurs every 3–4 months. However, there are no studies that explore the actual longitudinal relationship between blood glucose and HbA1c. Our hypothesis is that HbA1c should disproportionately reflect blood glucose measures from more recent days. In this example, we will demonstrate how to build a scalar-on-function regression model to explore the longitudinal relationship between intensively measured blood glucose over three months and the health outcome HbA1c, predict HbA1c, and showcase the ability of fPCA to recover a smooth curve underlying the intensively measured glucose data over three months for each individual.

#### Design

The parent study was a single-arm longitudinal observational study. Each patient was provided with a cellular-enabled scale and a smartphone-tethered wrist-worn activity tracker and glucometer. Daily self-measurements of weight, physical activity, and blood glucose data were collected over 6 months [35, 39]. Data were aggregated on a research platform.

#### Study participants

Sixty adult patients with were recruited from the Duke Family Medicine Center. Participants who were eligible were at least 18 years old, able to speak and read English, diagnosed with type 2 diabetes mellitus, prescribed to monitor their blood sugar at least weekly, on diabetes-related medication, and owned an Android or iOS smartphone.

## Measures

### HbA1c

HbA1c values closest to 3, 6 and 9-month follow-up dates were extracted from the electronic health record (EHR). In this study, we used the HbA1c value closest to the 6-month follow-up date as the outcome.

*Blood glucose*: Blood glucose was measured by glucometer for 6 months. Although the inclusion criterion only required measurement of blood glucose at least once a week, most patients measured their blood glucose more frequently, either daily or multiple times a day. As blood glucose varies depending on patient diet, there were 9 available labels when recording glucose data: before-breakfast, after-breakfast, before-lunch, after-lunch, before-dinner, after-dinner, after-snack, at midnight, and fasting. As fasting glucose values are more stable, the before-breakfast glucose value was most frequently measured among all meal labels. In this study, we only included the before-breakfast glucose measures.

### Statistical analysis

To understand the data structure, an R Shiny app was developed so each patients’ data visualization was easily produced. To build a HbA1c prediction model, we used all the samples that had HbA1c values at a 6-month follow-up time (outcome) and if they had any pre-breakfast measurements of blood glucose data in the previous 3 months. Duplicated measurements on the same day were removed. Sixteen of 60 participants were excluded because they either lacked before-breakfast glucose measurements within the past 3 months or had missing 6-month HbA1c values. The median number of before-breakfast measurements per person in the analytical dataset was 46 out of 91 daily measures (3 months). As 54% of before-breakfast values were missing, fPCA for sparse functional data was used to estimate the intensively longitudinal blood glucose data for each individual. The input data were centered by subtracting averages over all subjects. The smallest number of functional principal components were chosen such that over 95% cumulative variance of original data can be explained by the model. Scalar-on-function regression model was built to model the association between 6-month HbA1c and the longitudinal blood glucose data in the preceding 3 months. Adjusted ${R}^{2}$ was calculated to assess the goodness of fit for the model. In addition, mean squared error (MSE) and Spearman’s correlation coefficient from a leave-one-subject-out cross validation were obtained as evaluation metrics for the model predicting HbA1C values.

## Implementation

The implementation of fPCA and the subsequent regression model is straightforward by using off-the-shelf software as we shall demonstrate in this section. The R codes to apply fPCA on sparse blood glucose data using “face” package [33] is presented below:

# fpca on glucose data.

fit_face <- face.sparse(data = glucose_long, argvals.new=(-90:0), newdata = glucose_long, calculate.scores = T, pve = 0.95, knots = 35, center = F).

*face.sparse* is a R function to estimate covariance functions for sparse functional data. The argument “*data*” represents the sparse functional data frame in long format consisting of three columns: observation times, subject indices, and values of observations without missing values. “*argvals.new*” is the vector of complete observation times, which is 91 days in our case. To save the fitted fPCA values, we can let “*newdata*” equal to the original functional data. “*calculate.scores*” is used to specify whether scores of EFPCs need to be calculated. “*pve*” is set to 0.95 to indicate the number of EFPCs will be selected such that the proportion of variance explained is at least 0.95. We can specify the number of knots to better capture the curvatures of the longitudinal data by using “*knots*” for penalized splines. The option “*center*” was set to false, which means that the input functional data have been centered.

To fit the scalar-on-function regression on 6-month HbA1c using the eigen scores for the preceding 3 months glucose data, the following R codes are used:

scores <- fit_face$rand_eff$scores[,1:2])

FPCR <- lm(A1C ∼ scores).

alpha <- coefficients(FPCR) [1].

beta <- coefficients(FPCR)[2:3].

# calculate coefficient function back.

beta_t <- fit_face$eigenfunctions[,1:2] %*% beta.

Moreover, when the amount of missing data is small or after carrying out any missing data imputation, the “refund” R package can be used to build FPCR in one step:

FPCR2 <- pfr(A1C ∼ fpc(glucose_wide, pve = 0.95)).

plot(FPCR2) #plot coefficient function.

## Results

**Fig. 1:**
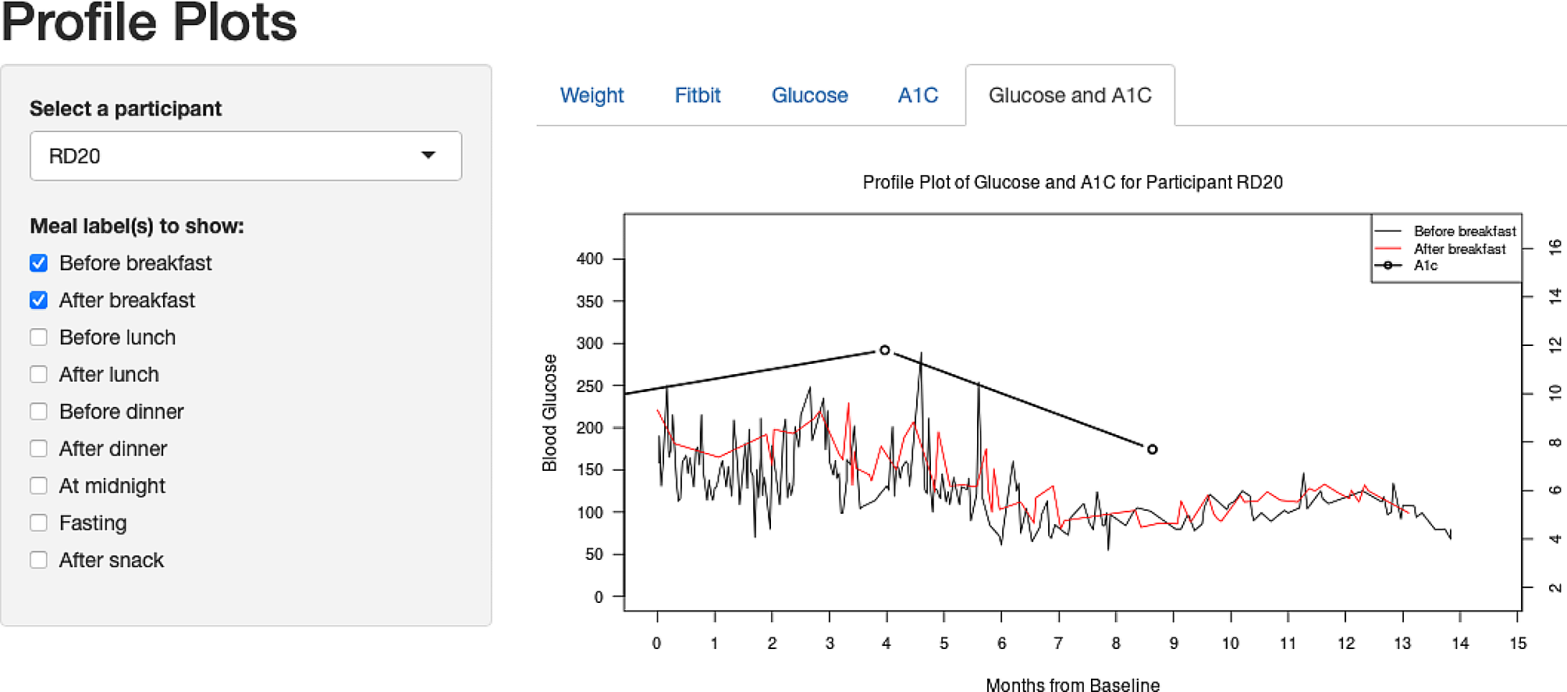
Screenshot of the user-friendly interface of the developed R shiny app (https://meilinj.shinyapps.io/ProfilePlots/)

A data visualization for one patient with all available HbA1c values from patients’ EHR and the trajectories of self-measured ILD on blood glucoses was provided in Fig. 1.

**Fig. 2:**
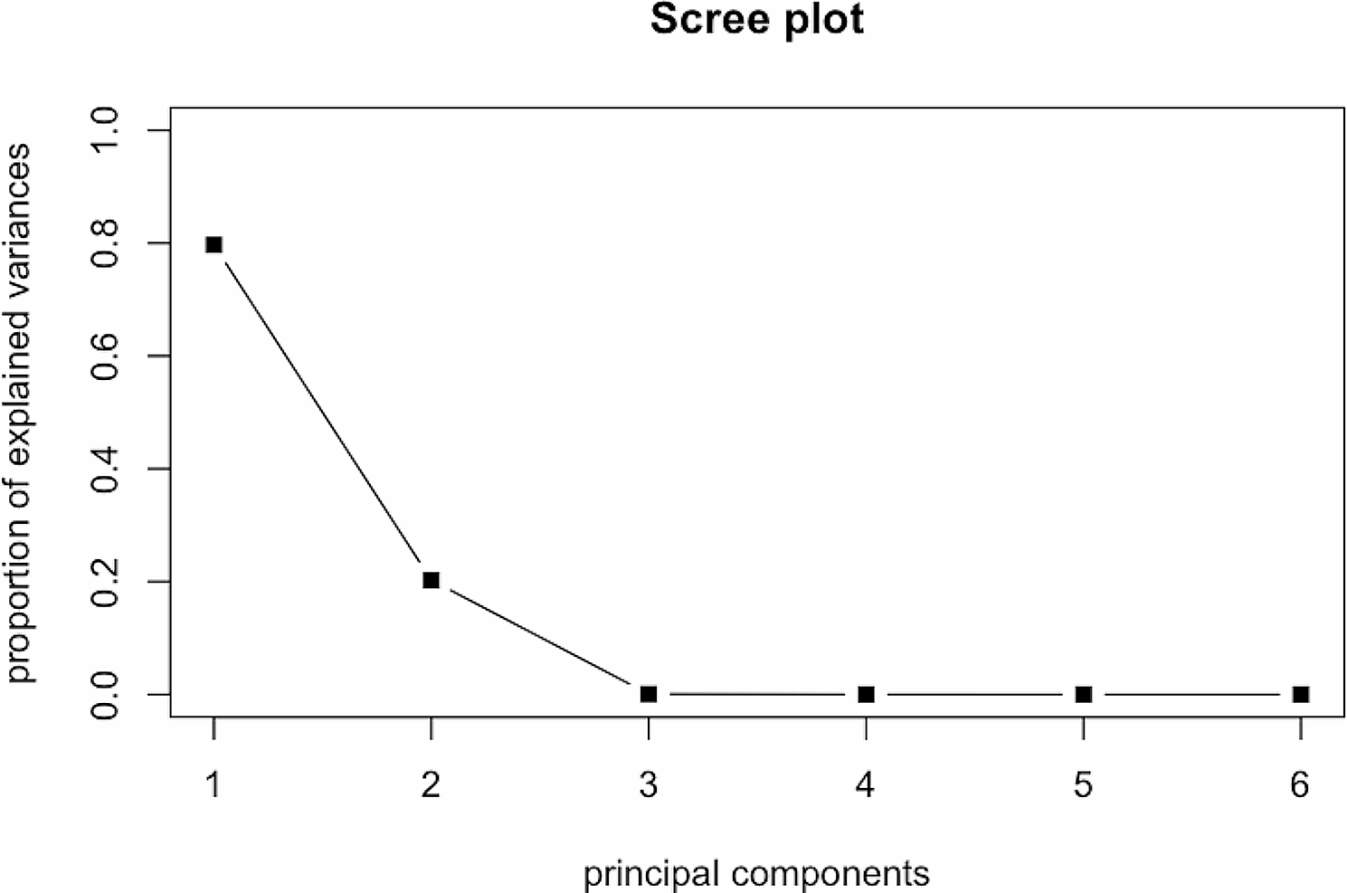
Scree plot to show the amount of total variance each functional principal component explained

**Fig. 3:**
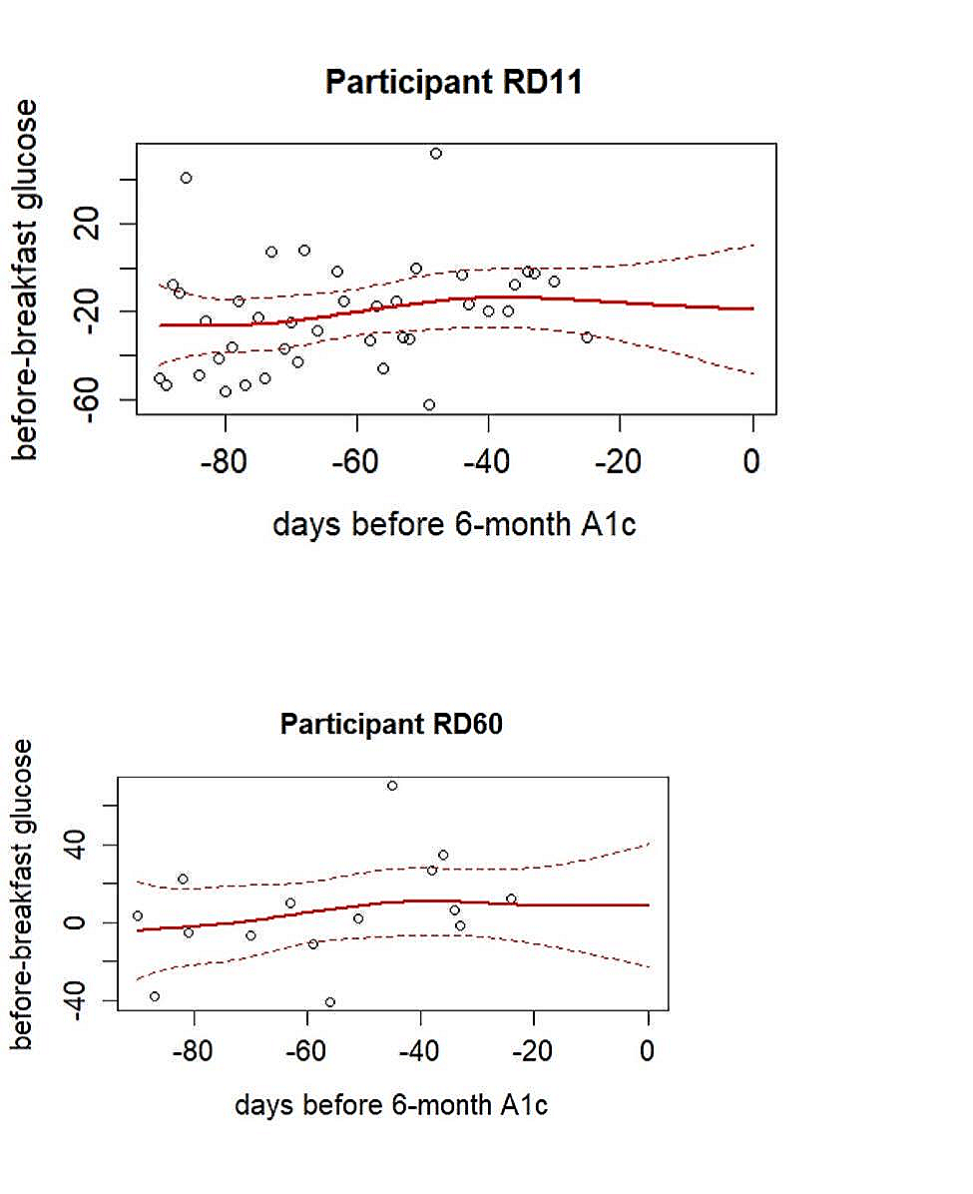
Observed data points (dots) and predicted glucose values (solid) with 95% CI (dashed) by fPCA

52 out of 60 patients had a 6-month HbA1c value, and 46 among them had at least one glucose measurement within the preceding 3 months. All these samples were used for fPCA and scalar-on-function regression model. For fPCA, two functional principal components (supplement figure S1) were chosen as they explain more than 99% of variance in the original glucose data (scree plot in Fig. 2). The observed and fitted trajectories of blood glucose from fPCA of two randomly selected participants were plotted in Fig. 3.

**Fig. 4:**
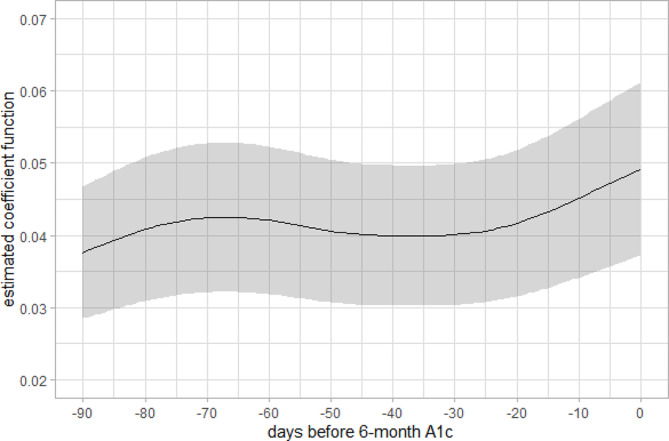
Estimated coefficient function $\widehat{\beta }\left(t\right)$ and its pointwise confidence band (shaded area) over time from functional regression model

**Fig. 5:**
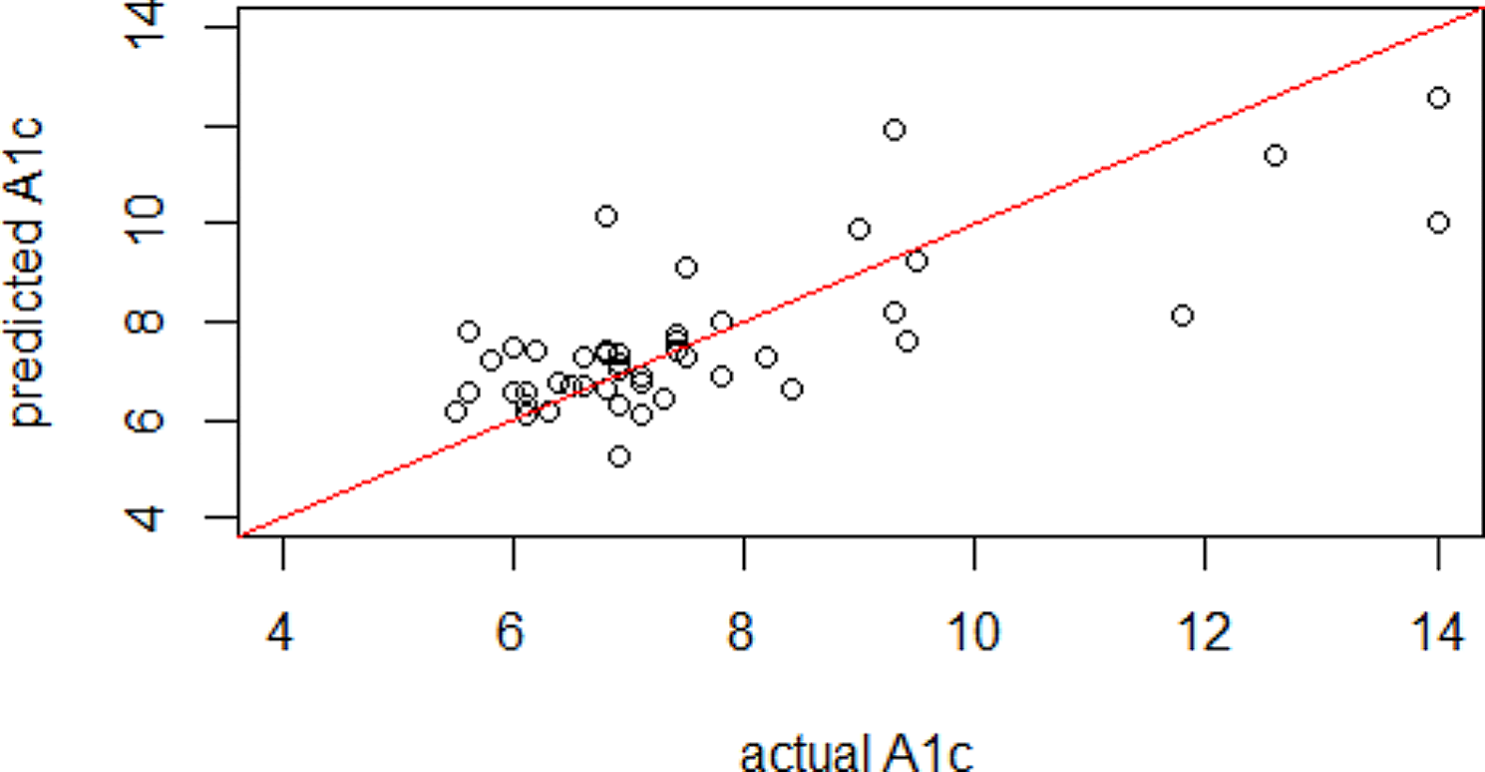
Predicted HbA1c and actual HbA1c values for all participants from leave-one-out cross validation. The red line is the 45-degree line to show equality

To build a functional regression model for the HbA1c, two statistically significant functional principal components were included in the regression model instead of 91 highly correlated daily blood glucose values over 3 months. The fitted model shows a significant relationship between intensive longitudinal blood glucose measurement and resulted HbA1c (*P* < 0.0001, ${R}_{adjusted}^{2}=0.61$). The estimated coefficient function $\widehat{\beta }\left(t\right)$, which describes how daily blood glucose measures over three months associated with HbA1c is shown in Fig. 4. As an evaluation of the prediction model, the mean squared prediction error (MSE) of 1.75 was obtained from a leave-one-subject-out cross validation. Figure 5 shows the predicted HbA1c against actual HbA1c values for each participant from cross validation. The spearman correlation between predicted and actual HbA1c values is 0.61.

## Discussion

Though often overlooked in medical research, fPCA offers an appealing tool to analyze mobile health device generated ILD, which are usually noisy and intermittently measured with missing values. It can capture subject-specific heterogeneity with i.i.d. principal component scores, which can be used by subsequent analysis, such as regression and other inference. It’s also a tool for exploratory data analysis, enabling data visualization of population trend and individual curves [21].

Compared to the traditional approach, using a functional regression model offers three main advantages. First, fPCA can directly utilize all data points from ILD, eliminating the need to choose a specific time window (e.g., weekly, or monthly) for averaging. Second, while a mixed effect model may also be able to assess longitudinal effect with a small number of time points and parameters, functional regression model provides a unique opportunity to assess complex dynamic relationship between ILD and the outcome in a functional form. In addition, fPCA is essentially a nonparametric estimation approach. It is more flexible to model complex trends in data which may be captured by a parametric model, such as linear mixed model [40]. Third, since functional regression model uses individual data points without averaging, it retains the granular information from ILD. Therefore, the model can potentially offer a better prediction especially when the actual longitudinal relationship between ILD and outcomes is not linear.

In the case study, functional regression model was used to predict HbA1c values for patients with type 2 diabetes based on their preceding three months’ blood glucose measurements. It also helped assess longitudinal association between blood glucose intensively measured over three months and HbA1c. In previous studies using response feature approach, different forms of average glucose values were used in analyses instead of longitudinal data points: average glucose over 3 months [41, 42], data from Korean population [43], average glucose from various meal labels [44], and weekly average glucose values [45]. Unlike the traditional response feature used in previous studies mentioned above, the functional regression approach was able to utilize all the available repeated measures directly from the glucometer. It does not require specifying a time interval to calculate average values, whether weekly, monthly, or every three months. Comparing to coefficients of a magnitude between 0.03 and 0.04 from previous research using response feature approach, the average of our estimated coefficient function over 3 months in our case study has a similar magnitude (Fig. 4). In addition, we were able to estimate the longitudinal association between three months daily blood glucose and future HbA1c (Fig. 4), an aspect traditional approach in previous studies could not address. Our results showed an overall increasing contribution from daily blood glucose values over three months in predicting HbA1c. While this exploratory analysis is only based on a small sample size, this overall trend does align with the understanding that HbA1c reflects red blood cell turnover and should disproportionately reflect blood glucose measures at more recent days. We also observed that fPCA regression has a comparable ${R}^{2}$ despite the fact that the sample size in the illustrative example is much smaller than other studies [41–45]. We also compared this with a regular regression model using the three months average blood glucose as predictor for our own data set. Since the actual longitudinal relationship between blood glucose and HbA1c is slightly increasing over time (Fig. 4), the ${R}^{2}$ for the two models are similar. However, we would anticipate that the ${R}^{2}$ for functional regression model is higher if there were other more prominent non-linear trends. Additionally our case study demonstrated that fPCA can recover the functional curve of blood glucose over time for each individual (Fig. 3). This would be not achieved if we were using traditional response feature approach.

Another advantage of the fPCA approach is its nonparametric nature, making it robust to model misspecifications. Moreover, it can be easily extended to models that include multiple functional predictors even if they are not measured at the same time. We can apply multivariate fPCA on all of the repeated ILD variables, such as blood glucose with each different meal-labels, daily measured weights, and exercise levels [34]. As the multivariate principal component scores are derived independently from the ILD, we can include them all together in the functional regression model without concerns about collinearity issues. This property is extremely useful for mobile health data as often we have multiple sources of mobile health data that could potentially help predict outcomes. Furthermore, since the functional regression model operates in two stages, the second stage can be viewed as a regular regression model with several principal components as predictors. This allows for the utilization of various other model-building techniques in the second stage to achieve a more comprehensive model. For instance, we could also incorporate other essential baseline factors for predicting health outcomes in addition to the ILD data.

Nevertheless, we will need to consider several factors applying the approach, as they may affect the power of assessing longitudinal association and the accuracy of the prediction model. Firstly, while there is no specific simulation study on sample size or a formally power analysis available, it is recommended to start with a reasonable sample size, and then use cross-validation to assess if the model estimates are stable. Secondly, although fPCA for sparse functional data is specifically designed for irregular repeated measure data, we may encounter some estimation issue when we have very scarce repeated measures around similar time points especially when the overall sample size is small. In practice, the true shape of the dynamic association is often unknown, necessitating careful examination of the observed data structure. Selecting the appropriate regularization, such as determining the number of knots in penalized splines for smoothing trajectories, is crucial to achieving optimal results. The “face” package typically employs penalized splines, which often require a relatively larger number of knots. This approach allows for a balance between model fitting and complexity [31]. Consequently, we can capture the nonlinear shape of the eigenfunctions (supplement figure S1), which serve as the basis for estimating beta, the coefficient function over time. Furthermore, if individual data measured over time tend to have high variation, more repeated measures will be beneficial in capturing the individual trend.

There are also a couple of limitations to this method. Firstly, as shown in Fig. 5, the predicted values tend to be biased downward for larger HbA1c values. Similar to other approaches, this is attributed to the limited number of samples with higher HbA1c values. Secondly, since the functional regression model is essentially a two-step approach, any bias in the functional principal component scores derived from the first stage could potentially affect the subsequent regression model. Joint model [46] for both outcome and the intensive longitudinal data together could be a valuable future direction to pursue. However, pursuing this direction would require considerable effort in developing methodologies, as there are limited existing tools available. Nonetheless, the simplicity provided by the two-step procedure provides an advantage in terms of computational ease [46].

## Conclusion

Given the availability of ILD generated from mobile health devices, FDA provides a promising tool to analyze data at a granular level in mobile health research. Compared to the response feature approach that averages data over time, FDA provides insights into trends and correlation information contained within intensive data, revealing hidden longitudinal patterns. Specifically, functional principal component regression is a useful tool for assessing dynamic longitudinal association between intensively repeated measurements and health outcomes, predicting health outcomes and recovering individual trajectories.

### Electronic supplementary material

Below is the link to the electronic supplementary material.

Supplementary Material 1

## Data Availability

The study is funded by NIH. The data is available for public upon request from corresponding author.

## Abbreviations

ILD: intensive longitudinal data
fPCA: functional principal component analysis
FDA: Functional data analysis
EHR: electronic health record
MSE: mean squared error
