# Supplementary material for: Predicting health outcomes with intensive longitudinal data collected by mobile health devices: a functional principal component regression approach
Source: BMC Med Res Methodol. 2024 Mar 17;24:69. doi: 10.1186/s12874-024-02193-7 (PMC10944610; doi:10.1186/s12874-024-02193-7)
Supplement: Supplementary file 1 — Supplementary Material 1 [file 12874_2024_2193_MOESM1_ESM.docx]

**Supplemental Materials:**

**Estimation process:**

In this section, we will focus on using scalar-on-function functional regression model to study the association between ILD and a scalar outcome. The model is formulated as

$Y_{i}=\alpha+\int X_{i}\left( t \right)\beta\left( t \right)dt+\epsilon_{i}$, (S1)

where $\alpha$is the intercept, $\beta\left( t \right)$ is the coefficient function of time t, which indicates level of importance of each measurement over time with respect to scalar outcome $Y$, and $\epsilon_{i}$ is the random error follows distribution of $N(0, \sigma^{2})$, $i=1,\ldots, n$. The biggest difference comparing to regular linear regression is that both the regressor $X_{i}\left( t \right)$ and coefficient function $\beta\left( t \right)$ are functions of time t. There are different ways to obtain unique estimation for $\beta\left( t \right)$ and fPCA-based method is the most commonly used one. The estimation process is done in two stages.

In the first stage, we need to represent intensively measured longitudinal data by smooth random functions $X_{i}\left( t \right)$. The fPCA approach models the data as smooth covariance functions with respect to different time points. The dimension of ILD is usually large given the large number of time points, and the correlations between these repeated measurements are high. fPCA uses *Karhunen*–*Loève* expansion to abstract orthogonal functions which represent the most prominent trends in variation of data. Assume that the random functions have been centered (Ramsay & Silverman, 1997, Crainiceanu & Goldsmith, 2010, Xiao et al. 2018). Every square integrable random function $X_{i}\left( t \right)$ can be represented by the expansion:

$$X_{i}\left( t \right)=\sum_{j=1}^{\infty} \zeta_{ij}\upsilon_{j}\left( t \right)$$

where $\upsilon_{j}\left( t \right)$’s are the eigenfunctions (principal directions) depending on the covariance function of $X_{i}\left( t \right)$, and $\zeta_{ij}=\int X_{i}\left( t \right)\upsilon_{j}\left( t \right)dt$ are the random scores associated with eigenfunctions. Eigenfunctions $\upsilon_{j}\left( t \right)$’s are arranged in a nonincreasing order such that the first component $\upsilon_{1}\left( t \right)$ represents the most significant trend deviated from the mean function since it explains the largest portion of variance. The score $\zeta_{ij}$ associated with each component describes how much $\upsilon_{j}\left( t \right)$ contributes to the i^th^ person’s subject-specific deviation from population mean function. In practice, although the expansion is perfect with countably many eigenfunctions, the underlying trajectory $X_{i}\left( t \right)$ for the i^th^ person, can be approximated with a good precision by choosing only the first $p$ eigenfunctions:

$X_{i}\left( t \right)\approx\sum_{j=1}^{p} \hat{\zeta}_{ij}\hat{\upsilon}_{j}\left( t \right),$ (S2)

where $\hat{\upsilon}_{j}\left( t \right)$ is the j^th^ estimated eigenfunction or estimated functional principal component (EFPC) of the covariance function of $X\left( t \right)$ among top $p$ important EFPCs, and $\hat{\zeta}_{ij}$ is the corresponding j^th^ estimated random score of i^th^ person, which is assumed to follow an independent and identically distributed (i.i.d.) normal distribution. Throughout the paper, the hat over a parameter indicates the parameter or function estimate. Before conducting fPCA step, we centered our data by subtracting estimated means over time (Ramsay & Silverman, 1997, Crainiceanu & Goldsmith, 2010.). The mean function estimator can be obtained by the average over all subjects, which is well-known consistent estimator for a fixed design as in our case (Crainiceanu et al. 2009). Given the sparse nature of and wiggly observed data, we have increased the number of knots to 35 for penalized splines in the “face” package, which was expected to better capture the curvatures of the trajectories. This enhancement helped to realize the nonlinear shape of the eigenfunctions (see supplement Figure 1) which were used as basis for estimating beta(t), the coefficient function over the time.

After representing $X_{i}\left( t \right)$ as a few principal components, in the second stage, we can proceed to the regression model part. It is assumed that the coefficient function $\beta\left( t \right)$ in equation (1) can be expanded by eigenfunctions such that

$\beta\left( t \right)=\sum_{j=1}^{p} \beta_{j}\upsilon_{j}\left( t \right)$. (S3)

Replacing $X_{i}\left( t \right)$ by a set of smooth curves according to (2), the regression model in equation (1) becomes a regular linear regression model shown as below

$Y_{i}=\alpha+\int\beta\left( t \right)\left( \sum_{j=1}^{p} \hat{\zeta}_{ij}\hat{\upsilon}_{j}\left( t \right) \right)dt+\epsilon_{i}$ ,

$=\beta_{0}+\sum_{j=1}^{p} \hat{\zeta}_{ij}\beta_{j}+\epsilon_{i}$， (S4)

where $\hat{\zeta}_{ij}$ is the functional score that was estimated from (2) and can be treated as the pseudo-covariates after dimension reduction. $\alpha$ is the intercept and $\beta_{j}=\int\beta\left( t \right)\hat{\upsilon}_{j}\left( t \right)dt$ is the estimated coefficient for the j^th^ component. Similar to a regular linear regression, we can obtain estimated intercept $\hat{\alpha}$ and coefficient for each component $\hat{\beta}_{j}$by least square estimate of regressing response $Y_{i}$ on estimated functional principal component scores $\hat{\zeta}_{ij}$:

$$\left[ \begin{aligned} \hat{\alpha} \\ \hat{\beta} \end{aligned} \right]={{(Z}^{t}Z)}^{-1}Z^{t}Y$$

Where $Z$ is the $n\times(p+1)$ design matrix $[\begin{matrix} 1 & \hat{\zeta} \end{matrix}]$. Recall that $\hat{\zeta}_{ij}=\int X_{i}\left( t \right)\hat{\upsilon}_{j}\left( t \right)dt$, then

$$Y_{i}=\alpha+\sum_{j=1}^{p} \hat{\zeta}_{ij}\beta_{j}+\epsilon_{i}$$

$$=\alpha+\sum_{j=1}^{p} \int{X_{i}\left( t \right)\hat{\upsilon}}_{j}\left( t \right)dt\beta_{j}+\epsilon_{i}$$

$$=\alpha+\int X_{i}\left( t \right)\sum_{j=1}^{p} \beta_{j}\hat{\upsilon}_{j}\left( t \right)dt+\epsilon_{i}$$

Comparing to equation (1), we can equate

$\beta\left( t \right)=\sum_{j=1}^{p} \beta_{j}\hat{\upsilon}_{j}\left( t \right)$ .

Now we can plug in the estimates $\hat{\beta}_{j}$back to estimate the original coefficient function $\hat{\beta}\left( t \right)$:

$\hat{\beta}\left( t \right)=\sum_{j=1}^{p} \hat{\beta}_{j}\hat{\upsilon}_{j}\left( t \right)$. (S5)


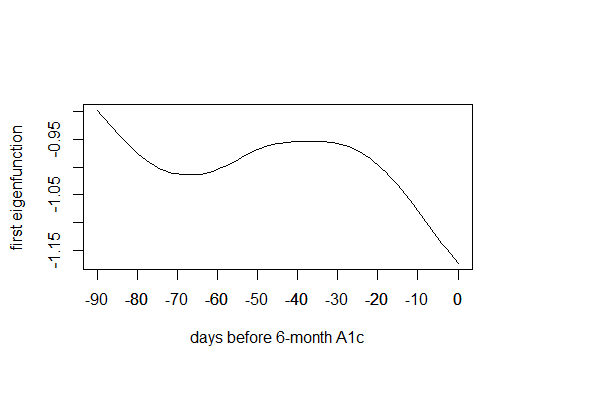

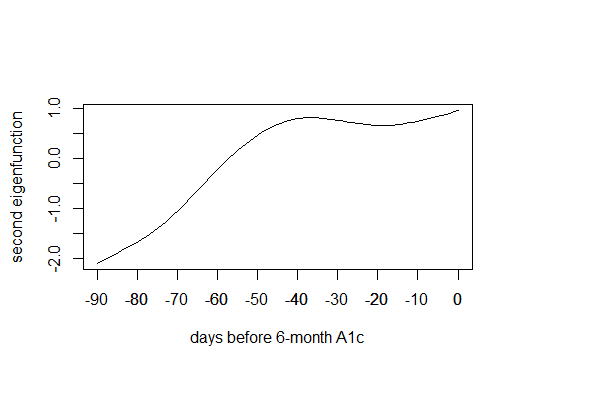


Figure S1 Principal component functions for fPCA.

**Results:**

Interpretation for beta (t): The estimated coefficient function characterizes the dynamic association between blood glucose over 90 days and HbA1c. In other words, the coefficient indicates the relative importance of each daily before-breakfast glucose measurement in contributing to the prediction of HbA1c. Specifically, a one mg/dL increase in blood glucose measurement would result in a beta (t) unit increase in HbA1c at time t. For example, a one mg/dL increase in blood glucose 90 days prior would result in a 0.038 unit increase in HbA1c. This relative contribution to future HbA1c increases from 90 days prior to 70 days prior to the HbA1c measurement, then gradually decreases, reaching 0.04 when it is 40 days prior, before starting to increase again 30 days prior to the HbA1c measurement
